# A Systematic Review of Case Reports of New-Onset Atrial Fibrillation in COVID-19 Patients

**DOI:** 10.7759/cureus.78938

**Published:** 2025-02-13

**Authors:** Rocio Barriga Guzman, Oluwaremilekun Tolu-Akinnawo, Toluwalase Awoyemi, Roseline Chima-Kalu, Oluwaseun Adeleke, Francis Ezekwueme, Joshua T Obarombi, Edwin Gwira-Tamattey, Oluwole Abib, Oladipo Odeyinka, Anderson C Anuforo

**Affiliations:** 1 Department of Internal Medicine, Advocate Illinois Masonic Medical Center, Chicago, USA; 2 Department of Internal Medicine, Meharry Medical College, Nashville, USA; 3 Nuffield Department of Women's and Reproductive Health, University of Oxford, Oxford, GBR; 4 Department of Pediatrics, University College Hospital, Ibadan, NGA; 5 Department of Internal Medicine, Sumy State University, Sumy, UKR; 6 Department of Internal Medicine, University of Pittsburgh Medical Center, Pittsburgh, USA; 7 Department of Internal Medicine, College Research and Innovation Hub, University of Ibadan, Ibadan, NGA; 8 Department of Internal Medicine, John H. Stroger, Jr. Hospital of Cook County, Chicago, USA; 9 Department of Internal Medicine, Piedmont Athens Regional, Athens, USA; 10 Department of General Medicine, College of Medicine, University of Ibadan, Ibadan, NGA; 11 Department of Internal Medicine, State University of New York (SUNY) Upstate Medical University, Syracuse, USA

**Keywords:** atrial fibrillation, cardiovascular implications, comorbidities, covid-19, management strategies

## Abstract

The severe acute respiratory syndrome coronavirus 2 (SARS-CoV-2) pandemic posed a significant global public health challenge, affecting millions of individuals. While some COVID-19 patients remain asymptomatic, others experience severe complications, including multiorgan failure and death. Emerging evidence indicates that COVID-19 is associated with substantial cardiovascular complications, notably an increased risk of arrhythmias, with atrial fibrillation (AF) being particularly prevalent among hospitalized patients. This review analyzes case reports of new-onset AF in COVID-19 patients, synthesizing data on patient demographics, comorbidities, clinical presentations, and outcomes. The cases reviewed indicate that affected patients were predominantly male, covered a broad age range, and frequently had underlying conditions such as hypertension, type 2 diabetes mellitus, and hyperlipidemia. The main outcomes observed included a high incidence of severe complications such as ischemic stroke, acute respiratory failure, myocarditis, and heart failure. Mortality rates were notably elevated among patients with COVID-19-related AF, particularly in those requiring intensive care or mechanical ventilation. The findings emphasize the significant cardiovascular burden of COVID-19, with a focus on its association with increased AF risk. By integrating case-based evidence, this review highlights the complex interplay between COVID-19 and AF, underscoring the need for early recognition and targeted treatment strategies to mitigate cardiovascular complications and improve patient outcomes in this vulnerable population.

## Introduction and background

The severe acute respiratory syndrome coronavirus 2 (SARS-CoV-2) outbreak triggered an unprecedented global health crisis, placing immense strain on public health systems worldwide [1]. While many individuals infected with the coronavirus disease (COVID-19) experienced mild or no symptoms, a significant proportion faced severe outcomes, including multi-organ failure and death [2]. Among the many systemic complications of COVID-19, its cardiovascular impact has become an area of increasing scientific interest. Notably, COVID-19 has been linked to a heightened risk of cardiac arrhythmias, with a particular emphasis on the development of atrial fibrillation (AF) in about 80% of patients with COVID-induced arrhythmias [3,4].

A comprehensive review of the relationship between COVID-19 and AF is necessary to deepen our understanding of the epidemiology, pathophysiology, and therapeutic strategies associated with this condition. Research has documented cases where COVID-19 either exacerbated pre-existing AF or contributed to the new onset of AF (the primary focus of our study), representing the commonest arrhythmias responsible for 80% of patients with arrhythmias [4], underscoring the importance of continuous investigation into this association. Despite the official end of the COVID-19 public health emergency, studying AF in the context of COVID-19 remains highly relevant. The persistent cardiovascular effects of the virus and the potential for AF to emerge as a prominent post-COVID condition highlight the need for further research and clinical vigilance [5,6]. This focus is critical for mitigating long-term cardiovascular morbidity and guiding effective management strategies for affected patients [7,8].

This review systematically evaluates the available evidence linking COVID-19 to AF, with a focus on characterizing the clinical features and pathophysiological mechanisms underlying new-onset AF in COVID-19 patients. The pathogenesis of AF in this context remains incompletely understood; however, several mechanisms have been proposed. Microvascular inflammation, fibrin deposition, endothelial dysfunction, and heightened platelet activation have all been implicated as contributors to AF in patients with severe COVID-19 [9,10]. These overlapping pathways may also explain the broader prothrombotic and inflammatory responses observed in the cardiovascular system during acute SARS-CoV-2 infection [9,10].

Furthermore, this systematic review examines case reports and case series to delineate the incidence, risk factors, and outcomes of AF among COVID-19 patients. It highlights the clinical management approaches and challenges faced in treating AF within this unique population. By synthesizing current findings, the review aims to address existing gaps in knowledge and lay the groundwork for future research into the intersection of viral infection and atrial arrhythmogenesis. The ultimate goal is to advance clinical practice by providing actionable insights that enhance the care of patients with COVID-19-related cardiac complications while informing strategies to mitigate the long-term burden of AF in post-COVID populations.

## Review

Methods

This study utilized a systematic review of case reports and case series to investigate the relationship between new-onset AF and COVID-19. A structured approach was followed, ensuring transparency, rigor, and reproducibility. The review adhered to qualitative synthesis guidelines, focusing on clinical features, pathophysiological mechanisms, incidence, risk factors, and outcomes associated with COVID-19-related AF.

Protocol Development

We followed the Alliance for Health Policy and Systems Research Guidelines for protocol development [11]. Key steps included are as follows: (1) refining the research question using the PEO (population, exposure, outcome) framework to focus on COVID-19 patients who developed new-onset AF; (2) designing a comprehensive search strategy with input from an information specialist to maximize sensitivity and specificity; (3) defining clear inclusion and exclusion criteria to ensure relevance and data quality; and (4) developing a standardized data abstraction form to facilitate consistent data extraction and synthesis.

This protocol was not registered in PROSPERO, as systematic reviews of case reports do not qualify for inclusion in its registry.

Search Strategy

A medical information specialist constructed the search strategy using Medical Subject Headings (MeSH) terms, subject headings, and synonymous keywords related to COVID-19 and AF. Databases searched included MEDLINE (via Ovid), Scopus, and Embase. The search covered all literature from database inception to March 17, 2024. Additional filters were applied to exclude non-peer-reviewed sources, gray literature, and theses. No language restrictions were imposed to ensure comprehensive data collection. Full details of the search strategy are available in the Appendices.

Eligibility Criteria

The inclusion criteria are as follows:

• Case reports and case series describing new-onset AF in COVID-19 patients confirmed via polymerase chain reaction (PCR) or rapid antigen testing.

• Electrocardiographic confirmation of AF within the same hospitalization.

• Studies providing detailed clinical characteristics, comorbidities, treatments, and outcomes.

The exclusion criteria are as follows:

• Non-human studies or those investigating arrhythmias other than AF.

• Reports with insufficient clinical details (e.g., missing electrocardiogram (ECG) confirmation).

• Retrospective or prospective cohort studies, review articles, and non-peer-reviewed literature (e.g., preprints, books, or book chapters).

This strict inclusion approach minimized biases and ensured a deep qualitative exploration of clinical presentations.

Study Screening and Selection

Title and abstract screening were conducted independently by TA, OTA, RBG, and FE, with full-text retrieval by TA, OTA, and OO. A full-text review was performed by TA, OTA, and AO, and discrepancies were resolved through consensus discussions. Rayyan (Rayyan Systems Inc., Cambridge, MA), an automation tool, was used to improve workflow efficiency [12].

Data Extraction

We developed a standardized data extraction form capturing the following: (1) study characteristics (authors, year of publication); (2) patient demographics (age, gender, number of cases); (3) comorbidities and pre-existing AF status (to differentiate new-onset AF from chronic AF); (4) diagnostic findings, including ECG results and pathological descriptions (e.g., cardiac biomarkers, autopsy reports, echocardiographic findings); (5) treatment modalities (anticoagulation, rate/rhythm control strategies, COVID-19-specific therapies); (6) clinical outcomes and endpoints, including the following:

• Primary outcomes: incidence of ischemic stroke, acute respiratory failure, myocarditis, and heart failure (HF) in patients with COVID-19-related AF.

• Secondary outcomes: mortality rates, ICU admission, need for mechanical ventilation, and length of hospital stay.

Inter-rater Reliability and Consensus Methods

To ensure data extraction consistency, five studies were independently reviewed to assess inter-rater reliability. Cohen’s kappa was calculated to measure agreement between reviewers. Disagreements were resolved by a third-party adjudicator (RCK) through a structured discussion and voting method.

Data Analysis and Synthesis

An inductive content analysis approach, based on Elo et al.’s framework [13], was applied in three phases:

Preparation phase - All reviewers convened to finalize eligibility criteria and search terms.

Organization phase - Data were coded thematically by RBG, EGT, and JO. Emerging patterns related to clinical characteristics, risk factors, and outcomes were identified. Thematic validation was conducted independently by multiple coders.

Reporting phase - A follow-up meeting was held to review thematic saturation, data consistency, and framework application. Findings were then synthesized into a structured summary table.

To enhance qualitative rigor, NVivo software (QSR International, Melbourne, Australia) was used for coding and thematic validation. No predefined reporting frameworks were used, but findings were structured based on emergent clinical patterns.

Results

Patient Demographics and Clinical Presentation

**Figure 1:**
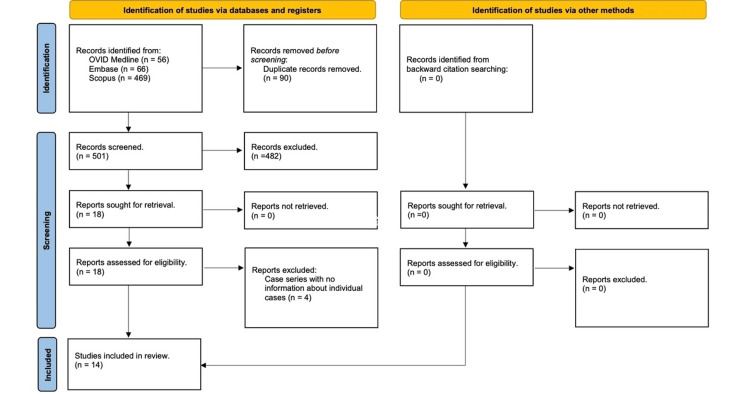
PRISMA flow diagram of our study PRISMA: Preferred Reporting Items for Systematic Reviews and Meta-Analyses Source: [11]

A total of 591 articles were identified from three databases: Ovid MEDLINE (n = 56), Embase (n = 66), and Scopus (n = 469) (Figure 1). After removing 90 duplicate records, 501 unique papers remained for screening. Initial title and abstract screening, guided by the predefined inclusion and exclusion criteria, resulted in the exclusion of 482 papers, narrowing the pool to 18 articles for further full-text evaluation. The screening process, detailed in the Appendices, involved careful assessment to ensure relevance and methodological rigor. Among the 18 reports retrieved, four were excluded due to insufficient case-level data (e.g., lack of COVID-PCR confirmation, unclear prior history of AF, missing ECG confirmation) in case series that did not provide individual patient information. Consequently, 14 studies [14-27] were deemed eligible and included in the final qualitative analysis. These studies were selected based on their alignment with the research question, adherence to inclusion criteria, and overall data quality, ensuring a robust and focused exploration of new-onset AF in COVID-19 patients.

The patient demographics from the selected studies revealed a higher prevalence of males among those with COVID-19-associated new-onset AF (Table 1). The age distribution ranged from 15 to 90 years, with a mean age of 55 years. Pre-existing cardiovascular risk factors were notable in this cohort: 16.7% (n = 4) had a history of hypertension, 12.5% (n = 3) had type 2 diabetes mellitus, and 8.3% (n = 2) had hyperlipidemia, consistent with the prevalence of CVD in the current literature [8]. Other comorbid conditions observed included obesity (n = 1, 4.2%), coronary artery disease (n = 1, 4.2%), tobacco use disorder (n = 1, 4.2%), pulmonary hypertension (n = 1, 4.2%), and sarcoidosis (n = 1, 4.2%). This diverse range of comorbidities underscores the complexity of managing new-onset AF in COVID-19 patients and highlights the necessity for tailored therapeutic strategies. These findings emphasize the multifactorial nature of AF development in the context of COVID-19 and its potential interaction with underlying cardiovascular and systemic conditions, offering insights for future clinical management and research. 

Figure 1 highlights the Preferred Reporting Items for Systematic Reviews and Meta-Analyses (PRISMA) flow diagram of our study.

Table 1 illustrates the patient demographics from the selected studies.

**Table 1:** Patient demographics from the selected studies

Gender	Frequency (n)	Percent (%)
Male	10	71.4
Female	4	28.5
Past medical history		
Hypertension	4	16.7
Type 2 diabetes	3	12.5
Hyperlipidemia	2	8.3
Pulmonary hypertension	1	4.2
Sarcoidosis	1	4.2
Obesity	1	4.2
Coronary artery disease	1	4.2
Smoking	1	4.2
Lung transplant	1	4.2
Non-significant/non-reported	8	33.4
Heart valve surgery	1	4.2
Presenting complaints		
Cough	6	15.0
Shortness of breath	5	12.5
Dyspnea on exertion	3	7.5
Fatigue	4	10
Fever	3	7.5
Lower extremity pain	2	5.0
Palpitations	2	5.0
Myalgia	2	5.0
Headache	3	7.5
Chills	2	5.0
Odynophagia	1	2.5
Altered mental status	1	2.5
Symptoms of atrial flutter	1	2.5
Symptoms of respiratory failure	1	2.5
Chest tightness	1	2.5
Diffuse erythema	1	2.5
Vomiting	1	2.5
Anosmia	1	2.5

Clinical Presentation

Among the patients studied, pulmonary symptoms were the most prevalent, affecting 32.1% of cases, followed closely by cardiovascular symptoms, observed in 28.6%. However, accuracy may be masked by an overlap between pulmonary and cardiovascular symptoms. Systemic and musculoskeletal manifestations were reported in 7.1% of cases, reflecting the broad spectrum of clinical presentations associated with COVID-19 and AF. Less commonly, a diverse range of other symptoms was identified, including endocrinologic, gastroenterologic, dermatologic, hematologic, head, eyes, ears, nose, and throat (HEENT), neurologic, and ophthalmologic manifestations, each reported in 3.6% of cases. This variability highlights the multifaceted impact of COVID-19 on different organ systems, necessitating a multidisciplinary approach to patient care.

Among the pulmonary symptoms, cough was reported by 15% (n = 6) of patients, while 12.5% (n = 5) experienced shortness of breath, a common and concerning indicator of respiratory involvement. Dyspnea on exertion (n = 3, 7.5%) and fatigue (n = 4, 10%) were also present, signaling the burden of respiratory compromise and systemic inflammation. Cardiovascular involvement, a central focus of this review, frequently presented with tachycardia, the most common physical sign, affecting 25% of cases. Hypoxia, indicative of impaired oxygenation, was noted in 20% of patients, underscoring the severity of pulmonary dysfunction associated with COVID-19-related AF.

Other notable clinical signs included respiratory distress, fever, hypertension, and tachypnea, each observed in 8.3% of cases. These findings emphasize the complex interplay between respiratory, cardiovascular, and systemic effects in patients with COVID-19 and AF. Table 1 provides a detailed breakdown of symptom prevalence, contributing to a nuanced understanding of the clinical presentation and guiding future management strategies for this high-risk patient population. The prominence of tachycardia and hypoxia as leading signs reinforces the need for vigilant cardiovascular monitoring and timely intervention to mitigate adverse outcomes.

Investigation Findings and Diagnosis

In this study, COVID-19 screening was performed using a combination of diagnostic methods in 42.9% of cases, reflecting a robust approach to confirming infection. PCR testing, the gold standard for COVID-19 diagnosis, was employed in 28.6% of cases, while rapid antigen testing was used in 14.3%. Some patients underwent multiple tests, with confirmatory PCR testing performed after initial rapid antigen testing to ensure diagnostic accuracy.

Every patient underwent an ECG evaluation, revealing AF with a rapid ventricular response (RVR) in 42.9% of cases. Additional ECG abnormalities included nonspecific ST-T wave changes (16.7%), QT prolongation (8.3%), and occasional premature ventricular contractions (4.2%), indicating varying degrees of myocardial and electrical instability. These findings underscore the direct cardiac implications of SARS-CoV-2 infection.

Inflammatory markers were elevated in 36.4% of patients, signifying systemic inflammation, a hallmark of severe COVID-19. Specifically, C-reactive protein (CRP) was elevated in 22.7% of cases, interleukin-6 (IL-6) in 13.6%, and ferritin in 18.2%, consistent with the hyperinflammatory response seen in COVID-19-related cytokine storms. Additionally, 9.1% of patients demonstrated other significant laboratory abnormalities, including elevated high-sensitivity troponin, indicating myocardial injury; increased N-terminal pro-b-type natriuretic peptide (NTproBNP), reflecting cardiac strain; coagulation abnormalities; and lymphopenia, consistent with immune dysregulation. These findings provide critical insights into the pathophysiological mechanisms linking COVID-19 to cardiovascular complications.

Imaging studies further highlighted the extent of pulmonary and cardiovascular involvement. Chest computed tomography (CT) and CT angiograms (CTAs) detected ground-glass opacities in 18.5% of cases, a characteristic feature of COVID-19 pneumonia. Chest X-rays revealed a range of patterns, including scattered airspace opacities in 14.8% of cases, interstitial infiltrates, and a hazy appearance in 8%. A combined analysis of chest CT and X-rays identified a unique COVID-19-related pattern in 7.4% of cases. Additional findings from chest X-rays included increased interstitial lung markings, small pleural effusions, vascular engorgement, cardiomegaly, and pulmonary edema, each occurring in 3.7% of cases. Chest CT also identified pericardial effusion in 3.7% of cases.

Abdominal and pelvic CTA imaging revealed acute limb ischemia and splenic infarcts, both occurring in equal proportions, highlighting the thrombotic complications associated with COVID-19. Notably, all patients with these thrombotic events had elevated D-dimer levels, and 66.7% were not on therapeutic anticoagulation prior to admission. Among those who developed ischemic complications, 50% had underlying risk factors such as new-onset AF or a prior history of thromboembolism. Management strategies included systemic anticoagulation in all cases, with the acute limb ischemia patient requiring interventional thrombectomy.

A single cardiac magnetic resonance imaging (MRI) study provided a detailed evaluation, demonstrating biventricular dysfunction and myocardial edema consistent with COVID-19-related myocarditis. Echocardiographic evaluations, summarized in Table 2, revealed significant cardiac dysfunction: 25% (n = 3) of cases exhibited reduced left ventricular (LV) systolic function, while right ventricular hypokinesis was also present in 25% (n = 3), often accompanied by pericardial effusion. Importantly, no cases of right ventricular dysfunction were definitively linked to pulmonary embolism, as all patients with RV impairment underwent CTA to rule out pulmonary thromboembolism.

**Table 2:** Overview of the echocardiographic findings EF: ejection fraction; LV: left ventricular; RV: right ventricular

Transthoracic echocardiographic findings	Frequency (n)	Percent (%)
Global impairment LV systolic function reduced EF	3	25.00
Preserve left ventricular EF	3	25.00
Moderate RV hypokinesis	2	16.67
Pericardial effusion	2	16.67
Severe left atrial enlargement	1	8.33
Heart failure with preserved ejection fraction/grade 1 diastolic dysfunction	1	8.33

Severe left atrial dilation was observed in 8.33% (n = 1) of cases, suggesting chronic pressure overload or structural remodeling. Additionally, manifestations of HF with preserved ejection fraction (HFpEF) or grade 1 diastolic dysfunction (G1DD) were noted in 8.33% of cases. While this proportion may seem lower than expected in an AF cohort, diastolic dysfunction was likely underestimated due to the technical limitations imposed by tachycardia, which can obscure accurate Doppler-derived diastolic parameters.

These findings (Table 2) collectively illustrate the diverse and complex cardiovascular impact of COVID-19, emphasizing the need for comprehensive diagnostic approaches and tailored management strategies. Table 3 provides a detailed overview of the echocardiographic findings, which further contextualize the relationship between SARS-CoV-2 infection and new-onset AF.

**Table 3:** Complications and outcomes observed in this cohort

Complications	Frequency (n)	Percent (%)
Acute respiratory failure	2	15.4
Ischemic stroke	2	15.4
Acute liver failure	1	7.7
Kidney infarction	1	7.7
Acute kidney injury requiring renal replacement therapy	1	7.7
Septic shock	1	7.7
Encephalopathy	1	7.7
Coagulopathy	1	7.7
Apnea	1	7.7
Spleen infarction	1	7.7
Hypertension	1	7.7
Outcomes		
Discharge	8	53.3
Improvement	4	26.7
Worsen outcome	2	13.3
Not available	1	6.7
Survival		
Alive	11	73.3
Deceased	2	13.3
Not available	2	13.3

Complications, Outcome, and Survival

The most common complications observed in this cohort were ischemic stroke and acute respiratory failure, each affecting 15.4% of the cases. These findings highlight the multifaceted impact of new-onset AF associated with COVID-19 on multiple organ systems. Ischemic stroke was reported in 16.7% (n = 2) of patients, reflecting the heightened thromboembolic risk linked to AF and COVID-19. Notably, anticoagulation strategies were considered in these cases, with one patient receiving therapeutic anticoagulation prior to the stroke event, while the other was not anticoagulated at the time of diagnosis due to concerns about bleeding risk. Additionally, splenic and kidney infarctions each occurred in 7.7% (n = 1) of patients, with some requiring renal replacement therapy, further illustrating the systemic vascular complications associated with the disease. Other complications recorded included septic shock, coagulopathy disorders, apnea, acute liver failure, and encephalopathy, emphasizing the broad and severe systemic effects of new-onset AF in the context of SARS-CoV-2 infection.

Cardiac complications were prominent among COVID-19 patients with new-onset AF. Myocarditis was the most frequent cardiac manifestation, occurring in 28.6% (n = 4) of cases, highlighting the inflammatory cardiac involvement seen with COVID-19. HF was observed in 21.4% (n = 3) of patients, underscoring the significant impact on cardiac function. Other cardiac complications included pericarditis, pericardial effusion, and non-ST-segment elevation myocardial infarction (NSTEMI), each affecting 7.1% (n = 1) of cases. The underlying mechanisms contributing to these myocardial complications likely involved a combination of direct viral injury to cardiomyocytes, systemic inflammation leading to cytokine-mediated myocardial damage, and stress cardiomyopathy triggered by the acute illness. These findings demonstrate the variety of cardiac pathologies triggered by COVID-19, suggesting a complex interplay of inflammation, thrombosis, and direct myocardial injury in the pathophysiology of new-onset AF.

Regarding patient outcomes and survival, an analysis of the case reports revealed that 53.3% (n = 8) of patients were discharged from the hospital after experiencing clinical improvement. A further 26.7% (n = 4) showed general health amelioration at discharge, while 13.3% (n = 2) experienced deteriorating conditions. Outcome data were unavailable for 6.7% (n = 1) of patients, primarily due to loss of follow-up or incomplete medical records.

Survival data from the reviewed case reports indicated that 73% (n = 11) of patients were alive as of the publication dates, while 13% (n = 2) had succumbed to their conditions. Among the fatal cases, the predominant complications leading to mortality included refractory cardiogenic shock, multisystem organ failure, and severe thromboembolic events. Long-term follow-up data post-discharge were limited as these were case reports. These findings (Table 3) underscore the variable prognosis associated with new-onset AF in COVID-19 patients and the need for continuous monitoring and tailored therapeutic strategies to mitigate adverse outcomes.

Treatment

In the studied patient cohort, rate control was the most common therapeutic approach for managing new-onset AF associated with COVID-19. Beta-blockers, particularly metoprolol and propranolol, were the preferred medications, and they were used in 53.33% (n = 8) of cases. These agents were selected due to their dual benefit of controlling ventricular rate and reducing myocardial oxygen demand, aligning with the inflammatory and hyperadrenergic states often observed in COVID-19 patients. Notably, calcium channel blockers, such as diltiazem and verapamil, were not widely used, primarily due to concerns about hypotension in critically ill patients. This highlights the importance of tailored rate control strategies in minimizing cardiac stress and preventing hemodynamic compromise.

Antiarrhythmic therapy was utilized in 40% (n = 6) of cases, with amiodarone being the primary agent. Amiodarone’s widespread use reflects its effectiveness in controlling both atrial and ventricular arrhythmias, particularly in patients with persistent or symptomatic AF when rate control alone was insufficient. Additionally, digoxin was used in 13.3% (n = 2) of cases, typically as an adjunctive therapy in patients with concomitant HF or hypotension, where beta-blockers were contraindicated or insufficient. Electrical cardioversion was performed in 13.3% (n = 2) of cases, and both patients had also received amiodarone prior to cardioversion, highlighting its role as part of a stepwise rhythm control strategy.

Anticoagulation therapy, aimed at preventing thromboembolic events, was administered in 46.67% (n = 7) of patients. The choice of anticoagulants included direct oral anticoagulants (DOACs) and low-molecular-weight heparin in 40% (n = 6) of patients, while warfarin was prescribed in 6.7% (n = 1) of cases, particularly for a patient with renal dysfunction requiring careful INR monitoring. The relatively moderate use of anticoagulation reflects a careful balance between mitigating thromboembolic risk and avoiding bleeding complications. Documented contraindications to anticoagulation included thrombocytopenia and active gastrointestinal bleeding in 13.3% (n = 2) of cases, while in other instances, the decision was based on clinician discretion due to concerns about bleeding risk in critically ill patients.

Treatment selection varied based on the severity of illness, with ICU patients more likely to receive rhythm control strategies, including amiodarone (55.6% of ICU patients) and electrical cardioversion, whereas non-ICU patients predominantly received rate control therapy with beta-blockers or digoxin. This trend reflects a more aggressive approach to rhythm management in critically ill patients with hemodynamic instability, while stable patients were managed conservatively to minimize adverse effects.

Table 4 provides a detailed breakdown of these therapeutic strategies, illustrating the diverse approaches required to manage AF in the context of COVID-19. These findings emphasize the importance of individualized treatment plans that account for the unique pathophysiological interactions between AF and SARS-CoV-2, ensuring both rate control and rhythm management are optimized while mitigating thromboembolic risk.

**Table 4:** Diverse approaches required to manage atrial fibrillation in the context of COVID-19

Treatment	Frequency (n)	Percent (%)
Rate control	8	53.33
Anticoagulation	7	46.67
Antiarrhythmic	6	40.00
Digoxin	2	13.33
Electrical cardioversion	2	13.33

Discussion

COVID-19 manifests with a wide range of symptoms affecting multiple organ systems, with significant impacts on the cardiovascular system [28]. Among cardiac complications, arrhythmias are commonly observed, including patterns such as S1/QIII/TIII, high-grade atrioventricular blocks, atrial tachycardia, atrial flutter, ventricular tachycardia or fibrillation, and pulseless electrical activity [15]. These findings underscore the profound influence of COVID-19 on cardiac electrophysiology. Notably, AF has emerged as a major cardiovascular complication, with its prevalence ranging from 19% to 21% in COVID-19 patients without pre-existing cardiovascular conditions to 36% in those with underlying heart disease, which is significantly higher than the general population without COVID at 1-2% [15, 29]. Although advanced age is a recognized risk factor for AF, it does not consistently predict the severity of COVID-19 outcomes [15].

The pathogenesis of COVID-19-induced AF is multifactorial and complex. Mechanisms linking COVID-19 to cardiovascular complications are both direct and indirect [30]. The interplay between the virus, host responses, and pre-existing cardiovascular comorbidities characterize this relationship [31]. Cardiac injury stems from viral interaction with host cells [30,31]. Autopsy reports reveal macrophage and lymphocytic infiltration, along with viral fragments in the endothelium of cardiac vessels, without evidence of viral replication [32]. Additionally, cytokine storms and immune dysregulation further drive inflammatory responses [33]. An imbalance between pro- and anti-inflammatory systems leads to erratic activation of inflammatory pathways, including catecholamine and IL-6 release, overwhelming the host’s ability to regulate these changes [34,35]. Catecholamine release increases heart rate and oxygen demand, predisposing patients to tachyarrhythmias such as AF, which impair cardiac function [30].

In COVID-19-induced AF, direct effects on cardiac myocytes disrupt cellular membranes and electrical signaling. Shared pathophysiological features, such as elevated CRP and IL-6, correlate with disease severity and prognosis [36-38]. Interactions with key receptors, including reduced angiotensin-converting enzyme 2 (ACE2), CD147, and sialic acid, exacerbate the cardiac response, promoting AF development [14,19,39-41]. Overactivation of the sympathetic nervous system and imbalances in electrolytes and acid-base homeostasis contribute to arrhythmogenesis. Increased calcium influx and diastolic calcium release via the ryanodine receptor (RyR) generate delayed afterdepolarizations (DADs) and action potentials, raising the risk of AF [42-44].

In our study, 14 patients were analyzed (Table 5), with 43% of AF cases diagnosed on presentation to the emergency department (ED), while 57% developed arrhythmias after one or more days of hospitalization. RVR occurred in 42.9% of initial AF cases. Presenting symptoms included cough, dyspnea, and fever; palpitations were reported in 5.7% of cases. Additional symptoms, such as chest tightness and light-headedness, were noted in 2.9%.

**Table 5:** List of case reports of new-onset AF in COVID patients AF: atrial fibrillation

Author	Title	Age	Gender	Complications	Survival (alive/dead/NA)
Gaine et al. [14]	COVID-19-Associated Myocarditis Presenting As New-Onset Heart Failure and Atrial Fibrillation	58	Male	Severe myocarditis, heart failure	Alive
Hajouli et al. [15]	A 29-Year-Old Man With COVID-19 Pneumonia, Heart Failure-Reduced Ejection Fraction, and Atrial Fibrillation With a Father and 2 Grandparents Who Were Positive for SARS-CoV-2 Infection	29	Male	Reduced ejection fraction, moderate right ventricular hypokinesis	Alive
Mandić et al. [16]	SARS-CoV-2 and* Plasmodium falciparum *CoInfection a Lethal Combination to the Heart	69	Male	Myocarditis, pericarditis, and acute heart failure	Alive
Seecheran et al. [17]	Atrial Arrythmias in a Patient Presenting With Coronavirus Disease-2019 (COVID-19) Infection	46	Male	None	Alive
Seres et al. [18]	Post-COVID Subacute Thyroiditis and Bronchiolitis in a Lung Transplant Recipient: A Case Report	53	Female	Subacute thyroiditis, decrease in forced expiratory volume in one second in spirometry	Alive
Al-Abbas et al. [19]	New-Onset Atrial Fibrillation and Multiple Systemic Emboli in a COVID-19 Patient	50	Male	Right occipital lobe subacute infarcts, left homonymous hemianopia, infarction of the inferior lobe of the left kidney, and hilum of the spleen.	Alive
Radwan et al. [20]	Disrupting the Electrical Circuit: New Onset Atrial Fibrillation in a Patient With Severe Acute Respiratory Syndrome Coronavirus 2 (SARS-CoV-2)	37	Male	None	Alive
Harhay et al. [21]	SARS-COV-2 Presenting as New Onset Atrial Fibrillation: A Case Report	90	Female	Hypoxia leading to intubation	NA
Mohamed et al. [22]	Multi-Triazole-Resistant *Aspergillus fumigatus* and SARS-CoV-2 Co-infection: A Lethal Combination	66	Male	Acute respiratory failure, acute kidney injury requiring continuous renal replacement therapy and vasopressor support due to septic shock	Dead
Sauer et al. [23]	Pericardial Effusion in Patients With COVID-19 Patients	60	Male	Moderate pericardial effusion	Alive
Kohli et al. [24]	Fulminant Myocarditis and Atrial Fibrillation in Child With Acute COVID-19	15	Female	Fulminant myocarditis	NA
Bouchlarhem et al. [25]	Brainstem Stroke: A Fatal Thromboembolic Event After New Onset Atrial Fibrillation During COVID-19 Infection: A Case Report and Literature Review	66	Male	Severe thromboembolic complications, including new-onset atrial fibrillation, ischemic stroke of the brainstem, and a fatal ventricular fibrillation	Dead
Carothers et al. [26]	Acetylcysteine for Treatment of Remdesivir-Induced Acute Liver Failure in COVID-19	68	Female	Significant increases in liver enzymes, coagulopathy, and encephalopathy, non-ST-elevation myocardial infarction	Alive
Zappa et al. [27]	Complications of SARS-CoV-2 Infection During Cardiac Rehabilitation: A Case Series	81	Male	Hypertension	Alive

Table 5 highlights the list of case reports used in our study.

AF impairs LV function, worsening pre-existing HF. Among critically ill COVID-19 patients, nearly 25% experienced a decline in LV systolic function, contributing to HF symptoms. In intensive care units (ICUs), AF affects approximately 10% of patients, with new-onset AF linked to poorer outcomes, including myocarditis, RV hypokinesis, pericarditis, and renal failure [17,45]. New-onset AF in COVID-19 pneumonia is associated with increased thrombotic risk, endothelial dysfunction, and oxidative stress, further highlighting its severe inflammatory and hypoxemic effects [46]. Consistent with prior studies, critically ill patients in our cohort demonstrated increased stroke risk, prolonged hospitalization, and higher mortality [47].

Data on rate vs. rhythm control in COVID-19-related AF remains limited. However, combining these strategies is associated with notable side effects [48]. Management focuses on restoring sinus rhythm to stabilize hemodynamics. In our cohort, beta-blockers (metoprolol and propranolol) and antiarrhythmics (amiodarone) were commonly used, tailored to underlying comorbidities. Cardioversion is recommended for patients with AF and RVR causing hemodynamic instability, with intravenous amiodarone preferred for rhythm control [49,50]. For stable patients, beta-blockers or non-dihydropyridine calcium channel blockers are preferred to mitigate QT prolongation risks [51]. COVID-19 also raises the risk of ischemic stroke, occurring in 2-6% of cases. This is attributed to a prothrombotic state and cardioembolic events [25]. Early stroke prevention with anticoagulation, guided by CHA₂DS₂-VASc scores, is essential, though COVID-19 treatments may interact with anticoagulants. For instance, antivirals such as lopinavir/ritonavir inhibit CYP3A4, increasing bleeding risks with non-vitamin K oral anticoagulants (NOACs), necessitating caution [52,53].

Beyond acute infection, AF may persist for months post-recovery. Longitudinal studies indicate a 44.2% recurrence rate within a year among patients with sepsis-related AF, analogous to COVID-19’s hyperinflammatory state, while recurrence rates are lower in patients without prior episodes [54].

Emerging management strategies for COVID-19-related AF include telehealth for remote monitoring and reducing hospital visits while maintaining effective care. Personalized medicine and technological innovations hold promise for refining prevention, detection, and treatment, emphasizing the need for ongoing research [49].

Limitations of study

While the focus on case reports and case series provides valuable clinical insights, it lacks statistical power and limits generalizability compared to randomized controlled trials. Small sample sizes further contribute to selection bias, making it difficult to draw definitive conclusions from a heterogeneous group of cases. Additionally, case reports and series are subject to publication bias, often emphasizing severe or unusual presentations, which may distort the perceived prevalence and severity of new-onset AF in COVID-19 patients.

Variability in symptoms, comorbidities, and treatment strategies complicate standardization and the development of universally applicable management guidelines. Missing or incomplete data in case reports also poses a challenge, as inconsistent reporting of diagnostic criteria, treatment responses, and follow-up outcomes can limit the reliability of findings. Moreover, the lack of assessment regarding the quality of included case reports and series raises concerns about the robustness of the data and the potential for overrepresentation of certain clinical patterns.

Finally, the absence of follow-up data in many cases prevents a comprehensive review of long-term outcomes, AF recurrence, and chronic cardiovascular effects. Future studies should aim for larger, more systematically collected datasets with rigorous methodological frameworks to enhance the validity and applicability of findings in broader clinical practice.

Future directions

While this article serves as a foundation for future research, larger prospective cohort studies and randomized controlled trials are needed to establish a more comprehensive understanding of the incidence, risk factors, and long-term outcomes of COVID-19-associated AF. Such studies would significantly enhance the evidence base to guide clinical practice. The exact mechanisms underlying COVID-19-induced AF remain unclear, necessitating further investigation into the molecular and cellular pathways involved. Research focusing on systemic inflammation, myocardial injury, thrombotic events, and direct viral effects on cardiac tissue could provide critical insights. The use of advanced biomarkers - such as troponins, inflammatory cytokines, and coagulation markers - along with imaging techniques like cardiac MRI and echocardiography may help identify early pathological changes and improve risk stratification.

Longitudinal studies are essential to evaluate the chronic cardiovascular effects of COVID-19-related AF, including recurrence rates, progression, and other long-term complications. Additionally, while this study explored demographic variations, future research should examine the impact of socioeconomic factors on COVID-19-associated AF. Investigating disparities in healthcare access, quality of care, and treatment outcomes among different socioeconomic groups could inform targeted strategies to reduce inequities and improve patient outcomes.

## Conclusions

This study offers an in-depth analysis of the complex relationship between COVID-19 and AF, highlighting key aspects of clinical presentation, diagnostic approaches, management strategies, and associated complications. By detailing the interplay between inflammatory responses, thromboembolic risks, and cardiovascular dysfunction, our findings contribute to the growing body of knowledge on COVID-19-related AF. Despite these insights, several gaps remain. The study underscores the need for more robust longitudinal studies to evaluate long-term outcomes, including rates of AF recurrence, post-discharge complications, and rehospitalization. Additionally, comprehensive mechanistic investigations are required to further elucidate the pathophysiological links between SARS-CoV-2 and AF, particularly the role of direct viral myocardial injury, systemic inflammation, and stress cardiomyopathy. Addressing these knowledge gaps will be essential for refining treatment strategies, optimizing anticoagulation protocols, and improving overall patient outcomes. Future research should focus on large-scale, multicenter studies to validate these findings and guide evidence-based clinical decision-making in the management of COVID-19-associated AF.

## Appendices

Search terms/search strategies

Embase = 66

('coronavirus disease 2019')/br OR ('Severe acute respiratory syndrome coronavirus 2') AND ('new-onset atrial fibrillation') AND 'case report' AND (1975:py OR 1976:py OR 1977:py OR 1978:py OR 1979:py OR 1980:py OR 1981:py OR 1982:py OR 1983:py OR 1984:py OR 1985:py OR 1986:py OR 1987:py OR 1988:py OR 1989:py OR 1990:py OR 1991:py OR 1992:py OR 1993:py OR 1994:py OR 1995:py OR 1996:py OR 1997:py OR 1998:py OR 1999:py OR 2000:py OR 2001:py OR 2002:py OR 2003:py OR 2004:py OR 2005:py OR 2006:py OR 2007:py OR 2008:py OR 2009:py OR 2010:py OR 2011:py OR 2012:py OR 2013:py OR 2014:py OR 2015:py OR 2016:py OR 2017:py OR 2018:py OR 2019:py OR 2020:py OR 2021:py OR 2022:py OR 2023:py OR 2024:py) AND 'article'/it AND ('case report'/de OR 'clinical article'/de OR 'human'/de)

OVID Medline = 56

COVID-19.mp. or exp COVID-19/ OR Severe acute respiratory syndrome coronavirus 2.mp. AND case report.mp. or exp Case Reports/ OR case series.mp. AND exp Atrial Fibrillation/ or new onset atrial fibrillation.mp. AND limit 1 to (English language and humans and yr="1860 - 2024")

Scopus = 469

( TITLE-ABS-KEY ( new AND onset AND atrial AND fibrillation ) OR TITLE-ABS-KEY ( atrial AND fibrillation ) AND TITLE-ABS-KEY ( severe AND acute AND respiratory AND syndrome AND coronavirus ) OR TITLE-ABS-KEY ( COVID-19 ) OR TITLE-ABS-KEY ( COVID-19 ) AND TITLE-ABS-KEY ( case AND report ) OR TITLE-ABS-KEY ( case AND series ) ) AND ( LIMIT-TO ( SUBJAREA , "MEDI" ) ) AND ( LIMIT-TO ( DOCTYPE , "ar" ) ) AND ( LIMIT-TO ( LANGUAGE , "English" ) ) AND ( LIMIT-TO ( EXACTKEYWORD , "Case Report" ) )
